# Parkinson’s disease in a patient with *GBA* and *LRRK2* covariants after acute hypoxic insult: a case report

**DOI:** 10.1186/s12883-023-03269-5

**Published:** 2023-06-10

**Authors:** Yuting Tang, Lijian Wei, Zhuohua Wu, Pingyi Xu, Mingshu Mo

**Affiliations:** Department of Neurology, the First Affiliated Hospital of Guangzhou Medical University, Guangzhou, China

**Keywords:** Parkinsonism, *GBA*, *LRRK2*, Hypoxic insult, Case report

## Abstract

**Background:**

The *glucocerebrosidase* (*GBA*) and *leucine-rich repeat kinase 2* (*LRRK2*) genes are associated with the risk of sporadic Parkinson’s disease (PD). As an environmental factor, hypoxic insults may impair dopamine neurons in the substantia nigra and exacerbate PD symptoms. However, covariants of *GBA* and *LRRK2* combined with hypoxic insults in clinical cases of Parkinsonism have not yet been reported.

**Case presentation:**

A 69-year-old male patient with PD and his relatives were clinically characterized and sequenced using the whole-exome technique. A novel covariant, *c.1448 T* > *C* (*p. L483P*, *rs421016*) on *GBA* and *c.691 T* > *C* (*p. S231P*, *rs201332859*) on *LRRK2* were identified in this patient who first developed bradykinesia and rigidity in the neck at one month after an acute hypoxic insult during mountaineering. The patient presented with a mask-like face, festinating gait, asymmetric bradykinesia, and moderate rigidity. These symptoms were treated with levodopa and pramipexole, resulting in a 65% improvement in the Unified Parkinson’s Disease Rating Scale (UPDRS) motor score. These parkinsonian symptoms persisted and developed with hallucinations, constipation, and rapid eye movement sleep behavior disorder. After 4 years, the patient exhibited a wearing-off phenomenon and died from pulmonary infection 8 years after disease onset. His parents, wife, and siblings were not diagnosed with PD, and his son carried *p. L483P* without Parkinsonism-like symptoms.

**Conclusions:**

This is a case report of PD after hypoxic insult in a patient carrying a covariant of *GBA* and *LRRK2*. This study may help us understand the interaction between genetic and environmental factors in clinical PD.

## Background

Genetic factors play an important role in the etiology of idiopathic Parkinson’s disease (PD). The *glucocerebrosidase* gene (*GBA*) encodes the lysosomal enzyme glucocerebrosidase, and its variant or dysfunction may increase the risk of Gaucher disease, Lewy body dementia, and PD [1]. Most *GBA* variant carriers in PD, such as *p. L483P*, showed earlier-onset, severe parkinsonian symptoms and an increased risk of dementia [1]. Another PD-related gene, *leucine-rich repeat kinase 2* (*LRRK2*), encodes a protein that interacts with the C-terminus of the parkin protein [2]. The *c.6055G* > *A* (*p. G2019S*) variant in *LRRK2* was identified to increase the risk of PD, Crohn’s disease, and leprosy [3, 4]. The PD patients with *p. G2019S* had asymmetric resting tremor, bradykinesia, and rigidity with a good response to levodopa [5]. Other variants in *LRRK2* in PD, such as *p. S231P,* are not yet clear*.* Acute hypoxic insult, classified as an environmental factor, may trigger or aggravate neurodegenerative diseases, but some studies have suggested that mild intermittent hypoxia may have neuroprotection in PD [6]. Acute hypoxic insult followed by mountain sickness may induce clinical symptoms, including headache, nausea, malaise, dizziness, insomnia, and cognitive dysfunction, but its relationship with PD is not clear [7, 8]. Here, we describe a Chinese patient carrying a covariant including *p. L483P* on *GBA* and *p. S231P* on *LRRK2* who showed Parkinsonism symptoms after an acute hypoxic insult for the first time.

## Case presentation

**Fig. 1:**
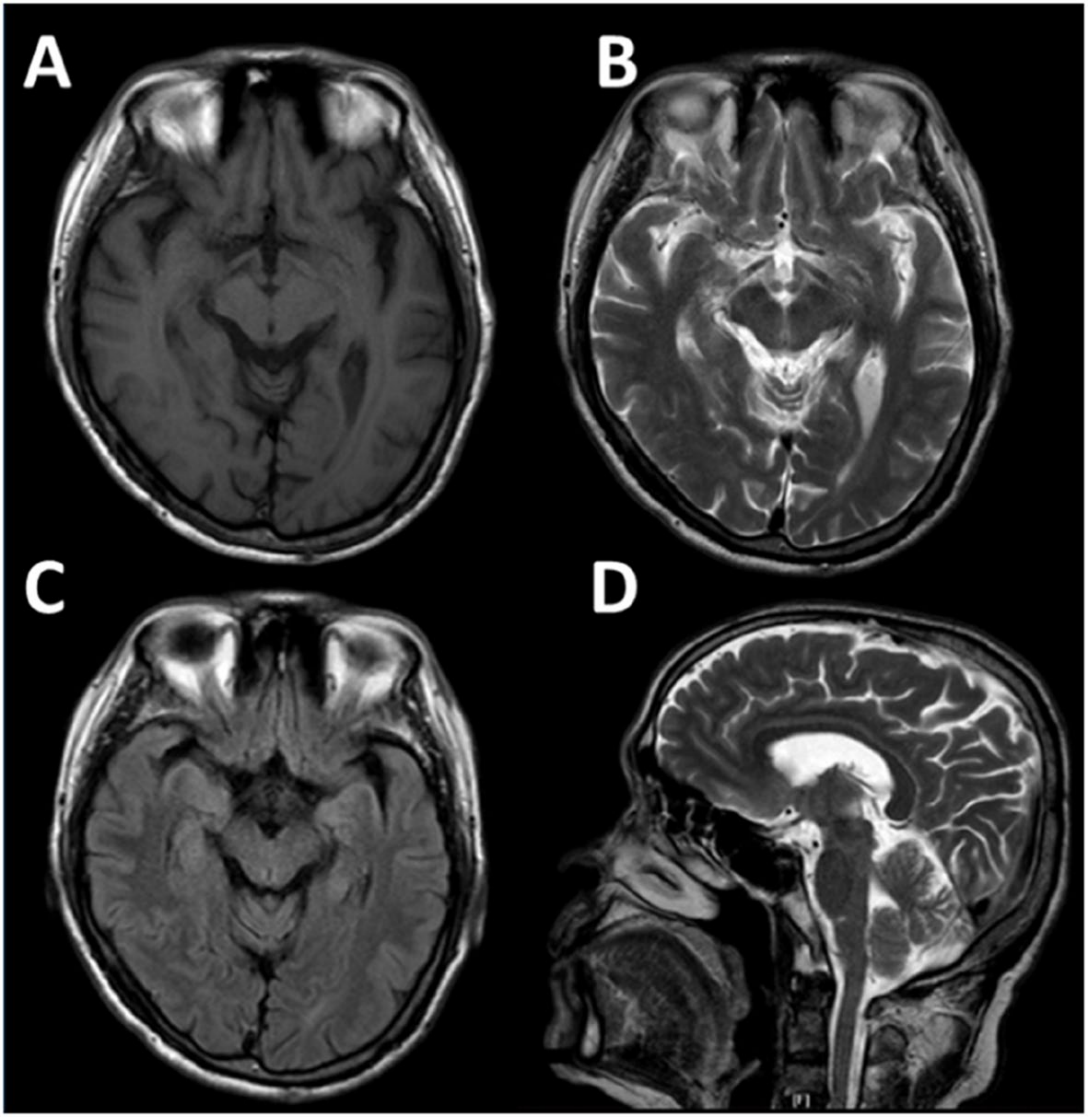
Brain structure of PD patients with magnetic resonance imaging. The MRI imaging performed transverse scans of T1-weighted (**A**), T2-weighted (**B**), T2-weighted (**C**), and sagittal scans of T2-weighted (**D**) images in 2017. No severe morphological changes in the brain were reported

The male patient, who had always lived in Foshan city with an elevation of approximately 13 m in China, had never gone to the plateau region and had not previously complained of any neurologic symptoms. In 2013, he was 69, traveled to the Tibetan Plateau which has an elevation of approximately 4,000 m, and stayed for 3 days without a high-altitude reaction. When climbing Mt. Namjagbarwa Hill with an elevation of approximately 6,700 m, he suffered a headache, vomiting, and dizziness after a strong cold wind. He experienced paroxysmal vertigo, headache, malaise, and nausea, accompanied by bradykinesia and rigidity in the neck, which was diagnosed as an acute hypoxia reaction by a local doctor. These symptoms were partly relieved after oxygen inhalation therapy. One month after he returned to a low altitude of approximately 13 m, vertigo disappeared, but the acute bradykinesia and rigidity symptoms continued. He went to the hospital and was found to have a mask-like face and bilateral mild cogwheel rigidity but no action or resting tremor, and he denied any cognitive or psychiatric symptoms. He was diagnosed with PD for the first time and his UPDRS-III total score was 37. The laboratory tests and anal sphincter electromyogram were normal. Brain magnetic resonance imaging (MRI, Fig. 1) and magnetic resonance angiography (MRA) scans revealed mild multiple stenosis in the bilateral posterior cerebral arteries (PCAs). The patient started drug treatment with levodopa and pramipexole, and his bradykinesia and rigidity symptoms were significantly improved with the UPDRS motor score increasing by 65%.

**Fig. 2:**
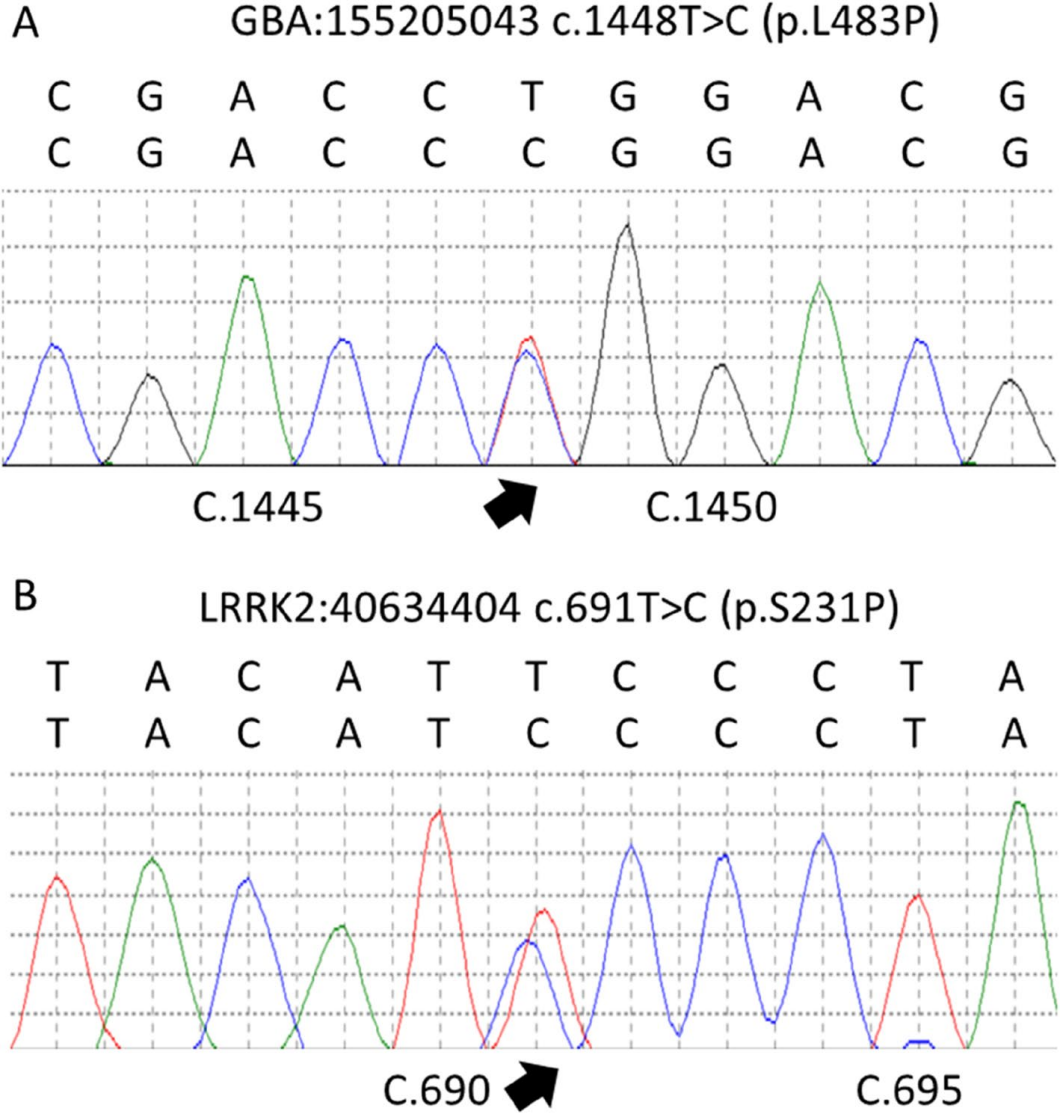
Genotype of PD by whole-exome sequencing. The whole-exome sequencing results showed that the Parkinsonism patient had covariants, including *c.1448 T* > *C* (*p. L483P*, rs421016) on *GBA* (**A**) and *c.691 T* > *C* (*p. S231P*, rs201332859) on *LRRK2* (**B**). The arrow points to the polymorphism variant site

After 4 years in 2017, he developed wearing-off phenomena with occasional foot dystonia. The dosages of levodopa and pramipexole were adjusted, and amantadine was added to relieve the worsening symptoms. After the treatment, his motor function improved. On a physical examination, he showed hypomimia, mild symmetric bilateral bradykinesia, mild bradykinesia, and festination gait. He had normal postural reflexes and no action or rest tremor. The UPDRS-III total score was 15. Some nonmotor symptoms emerged, including hallucinations, constipation, and rapid eye movement sleep behavior disorder (RBD), but no urinary dysfunction, depression, or cognitive decline. The laboratory tests were normal. The brain MRI and MRA scans showed multiple stenoses of the PCA as before. The whole-exome sequencing approach was used to detect PD-related genes in his family. His only child was 43 years old and healthy. The sequencing results showed that the patient carried a covariant including *p. L483P* on *GBA* and *p. S231P* on *LRRK2* (Fig. 2), and that his wife carried *p. S231P* and son carried *p. L483P* variants separately. His maternal and paternal families were of Southern Chinese ancestry, and his first-degree relatives had no family history of PD. After 8 years in 2021, he died from a pulmonary infection after aspiration.

Various computational methods were used to predict the pathogenicity of the *p. L483P* and *p. S231P* variants in PD, and showed that both of them were classified as variants of uncertain significance by ACMGG rules. The frequencies of variants were identified in 320 people with *p. L483P* out of 244,916 samples (1.31‰) and 10 people with *p. S231P* out of 245,502 samples (0.04‰) in the Gnomad database (http://gnomad.broadinstitute.org). The evaluation of *p. L483P* and *p. S231P* was performed on MutPred (score: probably damaging with 0.926 in *p. L483P*; benign with 0.229 in *p. S231P*; http://mutpred.mutdb.org/), SNPs&GO (score: disease with 0.844 in *p. L483P*; neutral with 0.127 in *p. S231P*; http://snps.biofold.org/), PROVEAN (score: deleterious with -4.995 in *p. L483P*; neutral with 0.004 in *p. S231P*; http://provean.jcvi.org/), PolyPhen-2 (score: 0.938 and 0.856, probably_damaging in *p. L483P*; 0.003 and 0.004, benign in *p. S231P*; http://genetics.bwh.harvard.edu/pph2/), Mutation taster (score: disease causing in *p. L483P*; polymorphism in *p. S231P*; http://www.mutationtaster.org/), and SIFT (deleterious in *p. L483P*; tolerated in *p. S231P*).

## Discussion and conclusions

Here, we report a case of a PD patient with variants of *p. L483P* on the *GBA* and *p. S231P* on the *LRRK2* genes after hypoxic insults. In China, people with the *GBA* variant (*p. L483P*) have been reported to have increased susceptibility to PD [9]. The *c.691 T* > *C* variant can induce *p. S231P* in the lrrk2 protein, but the pathogenicity of the *S231P* variant is not yet clear [9]. Little is known about the covariants of *GBA* and *LRR2* in sporadic PD combined with hypoxic insults. In this report, we describe a patient carrying (*p. L483P*) *GBA* and (*p. S231P*) *LRRK2* variants who suffered from a hypoxic insult and showed Parkinsonism symptoms with festinating gait, asymmetric bradykinesia, and moderate rigidity. This report suggested that interaction between covariants of *GBA* and *LRRK2* and hypoxic insults may have a relationship with PD risk.

*GBA* variants are common risk factors for PD. The OR (odds ratio) for any *GBA* variant is approximately 5.4 in PD [10]. PD patients carrying pathogenic *GBA* variants have approximately 5 years earlier onset, more advanced Hoehn and Yahr (H&Y) stage, and higher probability of suffering postural instability gait difficulty, but a similar response to levodopa treatment [10]. In the Chinese Han population, *p. L483P* is the most common *GBA* variant that increases the risk of early-onset PD with similar symptomology [11]. *GBA* is known to regulate the lysosomal-autophagy pathway and formation of Lewy bodies in PD pathogenesis [12]. The *p. L483P* variant may affect the function of GBA protein in PD according to pathogenicity prediction software [11]. In our report, we describe a PD patient with a covariant containing *p. L483P* on GBA and *p. S231P* on LRRK2. His initial symptoms were bradykinesia and rigidity, which were partly relieved by levodopa. Combined with the results of in-silico analyses, the *p. L483P* variant was suggested to increase the risk of PD. More studies are still needed to confirm the function of *p. L483P*.

The *LRRK2* gene encodes a serine/threonine kinase with GTPase activity, and its pathogenic variant is considered a risk factor for familial PD [2]. *LRRK2* is a multidomain protein kinase that includes an armadillo repeat domain from residue 150 to residue 510 and a serine/threonine protein kinase domain from residue 1879 to residue 2138 [2]. *p. G2019S* is a common pathogenic variant in the kinase domain, and may increase the kinase activity of *LRRK2*, which contributes to the progression of PD [5]. In this report, we found a rare heterozygous variant, *p. S231P*, which produces a serine to proline amino acid substitution at residue 231. In an epidemiological investigation, the carrier rate of *p. S231P* was 0.041‰ in 245,502 healthy samples from different countries [13]. A recent study based on an Asian population showed that one case carried the *p. S231P* variant in 1137 MSA patients and none in 619 healthy controls [13]. In the present report, we showed for the first time that a Chinese PD patient carried the *p. S231P* variant. The in-silico analyses suggested that the *p. S231P* variant had a weak association with disease. Based on the limited clinical data, more evidence is needed to explore the relationship between *p. S231P* and PD.

Acute mountain sickness is an illness related to acute hypoxic insult and always occurs after climbing to an altitude above 2,500 m without prior acclimatization [7]. The recoverable symptoms of acute mountain sickness include headache, malaise, nausea, dizziness, and insomnia [7]. *LRRK2* and *GBA* both contribute to regulating the oxidative stress-related signaling pathway [14, 15]. However, their relationship with environmental factors is unclear. Here, we showed that a PD patient carrying the *p. L483P* and *p. S231P* variants had a different clinical profile with unrecoverable symptoms after an acute hypoxic insult. The symptoms were partly relieved by levodopa and pramipexole. Combined with no cognitive and psychiatric symptoms at disease onset, these performances supported the diagnosis of PD but not Lewy body dementia [16].

In conclusion, we hypothesized that the covariants of *p. L483P* and *p. S231P* may impair the function of the oxidative stress-related signaling pathway, which makes acute hypoxic insult more serious. The underlying mechanism may help explain the interaction between genetic and environmental factors in PD and help to improve clinical treatments.

## Data Availability

The data and images used in this case report are available from the corresponding author on reasonable request.

## Abbreviations

GBA: Glucocerebrosidase
LRRK2: Leucine-rich repeat kinase 2
PD: Parkinson’s disease
MRI: Brain magnetic resonance imaging
MRA: Magnetic resonance angiography
RBD: Rapid eye movement sleep behavior disorder
PCA: Bilateral posterior cerebral arteries
UPDRS: Unified Parkinson’s Disease Rating Scale
OR: Odds ratio
H&Y stage: Hoehn and Yahr stage
