# Correction: Parkinson’s disease in a patient with *GBA* and *LRRK2* covariants after acute hypoxic insult: a case report

**DOI:** 10.1186/s12883-024-03916-5

**Published:** 2024-10-18

**Authors:** Yuting Tang, Lijian Wei, Zhuohua Wu, Pingyi Xu, Mingshu Mo

**Affiliations:** Department of Neurology, , Guangzhou, China, The First Affiliated Hospital of Guangzhou Medical University, Guangzhou, China

**Correction: BMC Neurol 23**,** 226 (2023)**.

10.1186/s12883-023-03269-5

Following publication of the original article [1], the authors reported an error found in the Funding section, funding number of “General Project of Basic and Applied Basic Research of Guangzhou Bureau of Science and Technology (2060206)” should be “202201020418”.

The original article [1] has been updated.
